# Effect of COVID-19 on Pet Food Bank Servicing: Quantifying Numbers of Clients Serviced in the Vancouver Downtown Eastside, British Columbia, Canada

**DOI:** 10.3389/fvets.2021.730390

**Published:** 2021-09-20

**Authors:** Marina Schor, Alexandra Protopopova

**Affiliations:** Faculty of Land and Food Systems, Animal Welfare Program, University of British Columbia, Vancouver, BC, Canada

**Keywords:** homelessness, food bank, companion animal, pets, human-animal bond, COVID-19

## Abstract

Previous research has focused on the benefits and difficulties of pet ownership in people, who are experiencing homelessness. However, many pet services, such as pet food banks, serve a more varied population of people. Furthermore, the effect of the COVID-19 pandemic has not been documented within the context of pet food banks. Vancouver's Downtown Eastside (DTES) population comprises a notable proportion of the city's overall population and has a high density of people who are experiencing financial hardships, but some of whom do not always experience homelessness. The purpose of this study was to gain an understanding of the number of clients and pets that are being serviced by a pet food bank, whether that has changed over time, and if it was impacted by the COVID-19 pandemic. We analyzed available attendance and service records from The British Columbia Society for the Prevention of Cruelty to Animals pet food bank between 2013 and 2020. We found that a median of 100 clients attended the food bank each week and that most of the companion animals serviced were cats (72.5%), then followed by dogs (25.2%), and rats (1.2%). Servicing was not consistent over time, with a weekly pattern of decreased attendance every fourth week of the month, which coincided with income assistance payments. This suggests that either servicing needs are decreased with income assistance or that the week of the month may present an access to care challenge. We also observed a decrease in the clientele attending in 2020 compared to previous years, suggesting an effect of COVID-19. Specifically, this trend was present for cats, rats, rabbits, and “other” companion animals, but not for dogs; the number of dog owners receiving services did not change in 2020, suggesting a difference between needed services in dog vs. other pet owners. The yearly trends shed light on the impact of COVID-19 on vulnerable populations, highlighting the need for additional support through times of crisis. Overall, the data show a complex relationship between pet service provision and other community issues and highlight the need to consider pet food banks within the greater social services networks.

## Introduction

The current global pandemic (COVID-19) has had a disproportionate impact on underserved populations (1–3). The pandemic has for instance, increased the level of food insecurity experienced worldwide, which serves to widen the gap between different socio-demographic groups (4–7). Furthermore, in the pandemic context, social work has become increasingly harder to perform due to government restrictions reducing in-person services, coupled with a lack of funding (8). Food banks are included in the social services that have been impacted by the pandemic, and certain cities have made active efforts to enhance offerings to support their populations (9). A deeper look into how the current pandemic has impacted access to social services such as that of pet food banks is paramount to ensure appropriate support is still available to populations in need. At the same time, research showed an increase in the strength of human-animal bonds during the COVID-19 pandemic (10–12), which all points to the importance of providing social structural support so that pet ownership can remain an option to those who benefit.

Research has demonstrated that owning companion animals while experiencing homelessness can have a myriad of mental health benefits (13, 14), and can help prevent the performance of self-destructive behaviors such as drug and alcohol misuse (15, 16). Furthermore, pet ownership in times of difficulty has been specifically shown to increase resiliency in vulnerable populations (17). Most of the current available data on pet-owning populations, who are experiencing financial hardship, are either restricted to those experiencing homelessness (i.e., living on the streets or otherwise unhoused), or focus on public perception of those populations (13, 17–19). However, populations experiencing hardship not only include those that are experiencing homelessness, but also involve people living in low-income housing, experiencing general housing instability, or who are “couch surfing,” for example (20–22).

**Figure 1:**
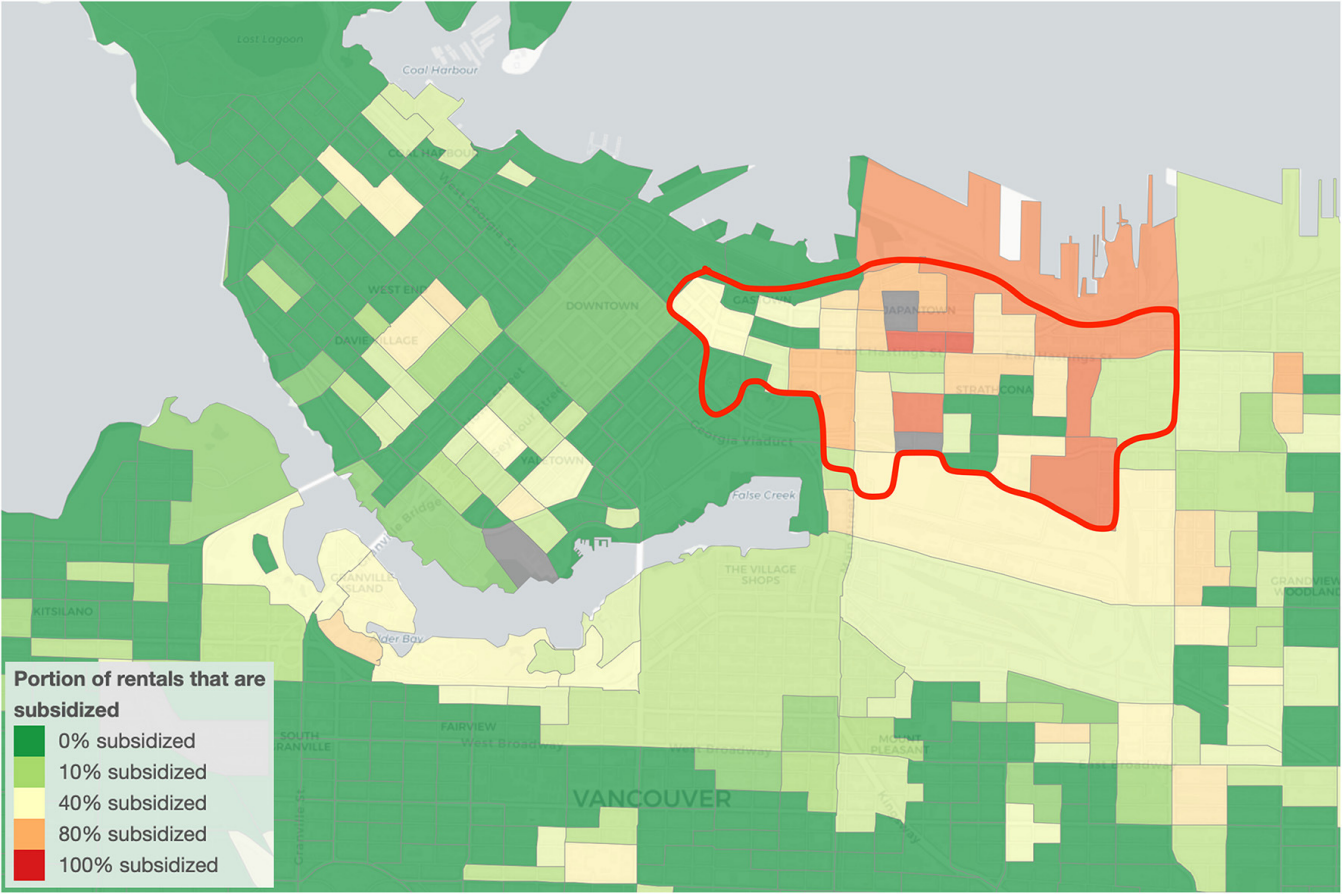
Partial map of Vancouver, B.C., Canada, with the red line outlining the approximate area of the Vancouver Downtown Eastside (DTES). The portion of rentals that are subsidized, taken from 2016 census data, are color-coded as an example of financial challenges of the area. Source: OpenStreetMap; mountainmath.ca/census.

According to previous census data, the Downtown population of Vancouver, British Columbia (B.C.), Canada, comprises over 10% of the city's general population and is heavily marked by special challenges. For instance, 30% of the indicated downtown Vancouver population reports experiencing limitations in their day-to-day activities, often due to disabilities, and that proportion increases to over 50% when specifically looking at Indigenous or elderly populations. The area's population is composed in its majority of male-identified individuals, with female-identified people composing 47% of the population, as opposed to 51% in Vancouver, in general (23). Further, the age profile of residents of the Downtown area does not contain a large number of school-aged children, with those in their 30's making up a large proportion of the population (23). In terms of housing, the Downtown area contains a significantly larger proportion of apartment buildings than does the general city of Vancouver, with about 97% occupancy in the area being in apartment units as opposed to 61% occupancy of apartments in the city, in general. Further, only 3% of homes are semi-detached houses or row houses or duplexes. Last, 94% of homes in the area are composed of two bedrooms or less (23). An unknown proportion of people are experiencing housing challenges in downtown Vancouver, and over 25% of that population currently lives below the poverty line (24, 25). The Downtown area also presents a higher proportion of people living alone than that of the general city, at 31% as opposed to 18% for Vancouver. Specifically, the proportion of seniors who live alone in the area is high, sitting at 37% as opposed to 29% for Vancouver in general (23). The area within downtown Vancouver that houses and serves ~20,000 people with many experiencing financial hardships is generally regarded as the Downtown Eastside (DTES; Figure 1), which is ~4 km^2^ and comprises the neighborhoods of Gastown, Chinatown, and Strathcona. A study in the area which focused on drug usage found that 26% of the population examined had an overdose or “life threatening event,” and that 3% passed away between October 2015 and January 2019 (26). Anecdotally, the prevalence of companion animals in the DTES of Vancouver is high, however metrics of that population are lacking.

Research has demonstrated that the COVID-19 pandemic has had a disproportionate effect on populations in which opioid usage is high, causing an increase in depression, anxiety, loneliness, and frustration (27). To help address these challenges, government and non-profit organizations make several social support services available in the area (28–30), such as food banks, harm reduction and education centers, and emergency shelters. However, many community services were greatly reduced due to physical distancing requirements. For example, a safe injection site decreased its capacity by 75% resulting in only 6 available stalls (31).

During the pandemic, most veterinary services continued to operate but through virtual appointments and/or with only the animal admitted into the facility. The B.C. Society for the Prevention of Cruelty to Animals (SPCA) was deemed an essential service and thus was allowed to remain open, providing sheltering and veterinary services to Vancouver's pet population. The same was true for “Charlie's,” the pet food bank run by the BC SPCA in the DTES area. While at times the pet food bank had fewer available staff, the availability of pet food to the DTES residents throughout the pandemic was overall consistent (32).

We aimed to provide data on the effects of the COVID-19 pandemic on pet food servicing by analyzing past data records from a pet food bank offered by the BC SPCA, which operates in the DTES area (33). Our purpose was to examine services provided to the pet owning DTES population with the goal of characterizing it and to understand whether supportive servicing in the area has been consistent across time. We hypothesized that COVID-19 would affect the numbers of clients serviced. Furthermore, we suspected that the types of services needed (i.e., companion animal species needing services) may differ in a population that is disadvantaged, but not necessarily experiencing homelessness, compared to previously published literature on pet food banks.

## Methods

### Data Collection

We collected records from BC SPCA pet food bank “Charlie's” which operates every Thursday from 10 a.m. to 12 p.m. at a designated location in Vancouver's DTES. The data contained information regarding how many people and how many companion animals were serviced each week of the food bank. Services provided (e.g., nail trims) were also counted in addition to food and litter distribution but were not included in our analysis due to a lack of record-keeping consistency throughout the years. Thus, we refer to “servicing” in this paper, but are only describing pet food and cat litter provision. We aimed to collect records from 2013 to 2020, however 2015 and 2016 records were not found and thus were not included in our analysis. Paper records for 2013 and 2014 were digitized and combined with 2017, 2018, 2019 and 2020 records into a central database. The University of British Columbia Behavioral Research Ethics Board approved all research activities (H20-03807).

### Data Analysis

Data curation and analysis were conducted using RStudio Team, 2020 (34). The descriptive data were first analyzed through the calculation of the number of each species of companion animals serviced as a proportion of the total number of animals. Further, the median number of animals and people in attendance each week, as well as interquartile ranges were calculated; this was done as a median for all years, for each year individually, as well as for the combined data for each week of the month. The data were analyzed by conducting a Shapiro-Wilk test for each of the different species to check for normality, which determined that most of the data were normally distributed (all *p* < 0.05, except the quantity of dogs by event was at *p* = 0.058). A total of 12 Kruskal-Wallis one-way analyses of variance were conducted, comparing each group (humans, cats, dogs, rats, rabbits, and “other” animals) in turn by both the year, as well as the week of the month during which the food bank took place. For the comparisons that were statistically significant (*p* < 0.05), Dunn Test *post-hoc* analyses were conducted.

## Results

### Descriptive Data

**Figure 2:**
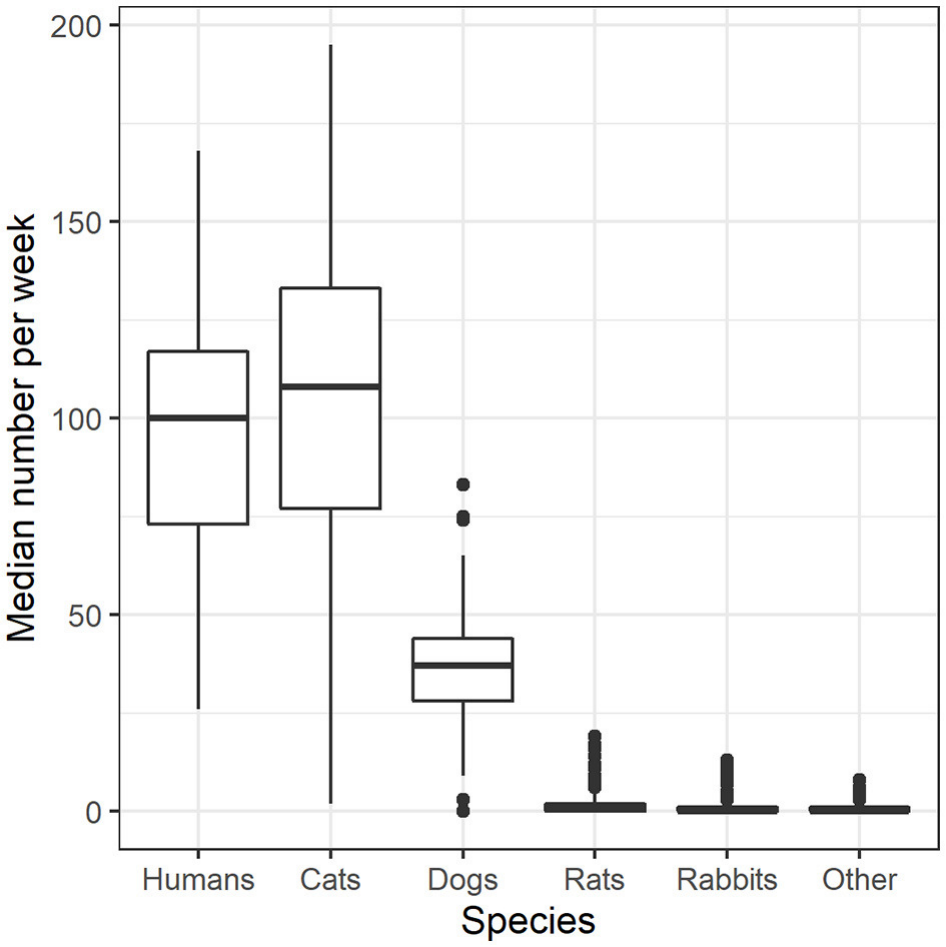
Median number of clients per week for all years combined. Medians and IQRs are shown.

The companion animal population serviced was comprised of mostly cats (72.5%), dogs (25.2%), rats (1.2%), rabbits (0.6%), and “other” animals (0.5%). The “other” category contained a vast array of animals such as birds, hamsters, guinea pigs, and chinchillas. Each week, a median of 100 people [interquartile range [IQR] = 44] were serviced by the pet food bank. Further, a median of 108 cats (IQR = 56), 37 dogs (IQR = 16), 1 rat (IQR = 2), 0 rabbits (IQR = 1), and 0 “other” animals (IQR = 1; Figure 2).

### Yearly Trends

**Figure 3:**
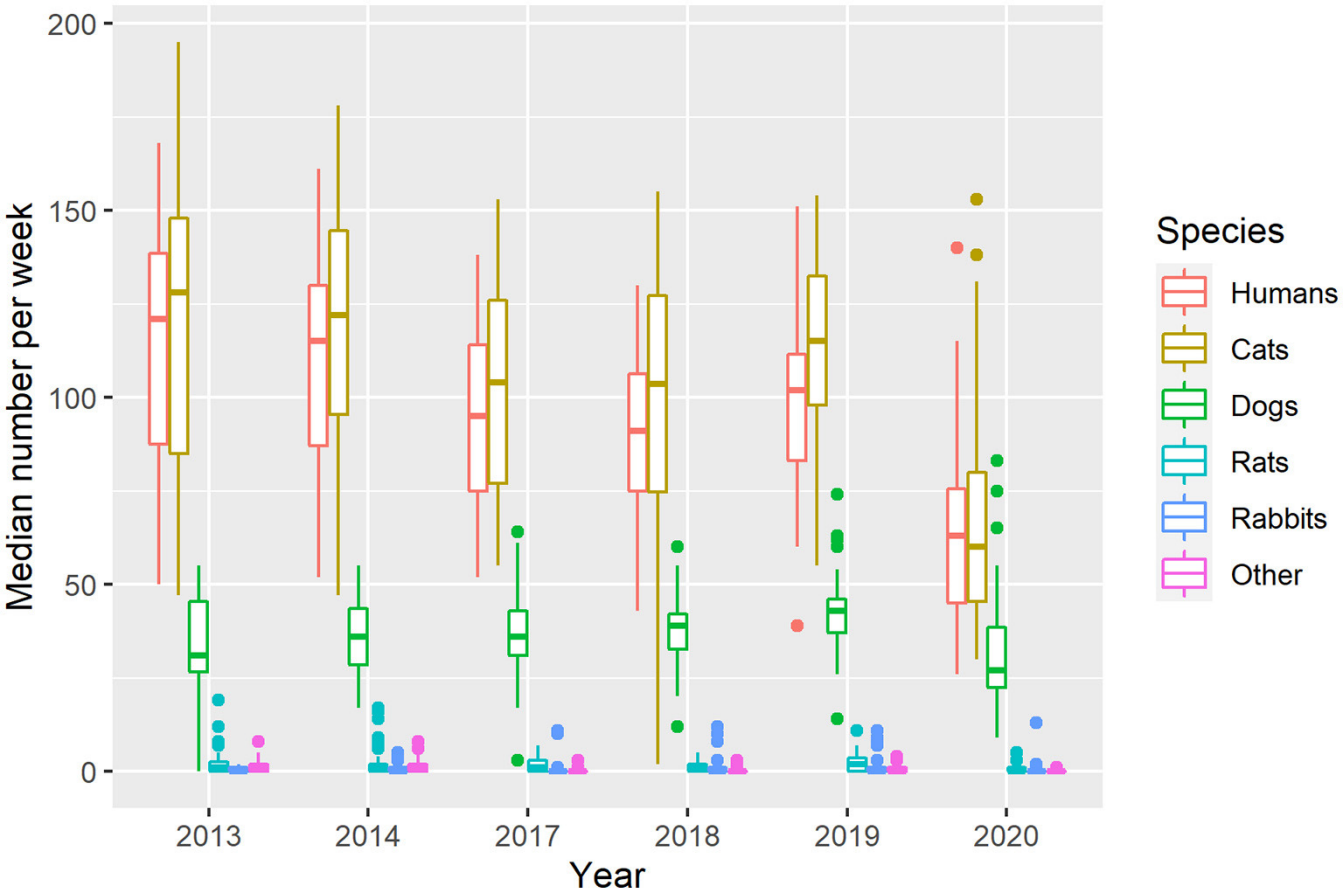
Median (IQR) number of clients per week by year.

The number of clients and animals serviced was different across sampled years [humans: H(5) = 72.9, *p* < 0.05; cats: H(5) = 63.9, *p* < 0.05; dogs: H(5) = 28.3, *p* < 0.05; rats: H(5) = 15.6, *p* < 0.05; rabbits: H(5) = 13.9, *p* < 0.05; “other”: H(5) = 67.7, *p* < 0.05; Figure 3].

More people were serviced in 2013 (median = 121, IQR = 51, *p* < 0.05), 2014 (median = 115, IQR = 43, *p* < 0.05), 2017 (median = 95, IQR = 39, *p* < 0.05), 2018 (median = 91, IQR = 31.25, *p* < 0.05), and 2019 (median = 102, IQR = 28.5, *p* < 0.05) when compared to 2020 (median = 63, IQR = 30.5, *p* < 0.05).

The number of cats who received pet food provisions weekly was higher in 2013 (median = 128, IQR = 63, *p* < 0.05), 2014 (median = 122, IQR = 49, *p* < 0.05), 2017 (median = 104, IQR = 49, *p* < 0.05), 2018 (median = 103.5, IQR = 52.5, *p* < 0.05), and 2019 (median = 115, IQR = 34.5, *p* < 0.05) when compared to 2020 (median = 60, IQR = 34.5, *p* < 0.05).

The number of dogs, however, was only statistically significantly lower in 2020 (median = 27, IQR = 16, *p* < 0.05) than in 2019 (median = 43; IQR =9, *p* < 0.05). It was equivalent to that of 2013 (median = 31, IQR = 19, *p* < 0.05), 2014 (median = 36, IQR = 15, *p* < 0.05), 2017 (median = 36, IQR = 12, *p* < 0.05), and 2018 (median = 39, IQR = 9.25, *p* < 0.05; Figure 3).

The number of rats in 2020 (median = 0, IQR = 1, *p* < 0.05) was equivalent to that of 2013, 2014, 2017, and 2018 (medians = 1, IQRs = 2.5, 2, 3, 2, respectively, all *p* < 0.05). However, it was lower than that of 2019 (median = 2, IQR = 3.5, *p* < 0.05). No statistical difference was observed in rabbit numbers between 2020 (median = 0, IQR = 0, *p* < 0.05) and any other year (medians = 0, IQR's = 1, 1, 0, 1, 1, 0 for 2013, 2014, 2017, 2018, and 2019 respectively, all *p* < 0.05; Figure 3).

The population of “other” animals was equivalent in 2013 (median = 1, IQR = 2, *p* < 0.05) and 2014 (median = 1, IQR = 2, *p* < 0.05). These were both higher than the populations observed in 2017 (median = 0, IQR = 0, *p* < 0.05), 2018 (median = 0, IQR = 0, *p* < 0.05), 2019 (median = 0, IQR = 1, *p* < 0.05), and 2020 (median = 0, IQR = 0, *p* < 0.05), which were equivalent to each other (Figure 3).

### Weekly Trends

**Figure 4:**
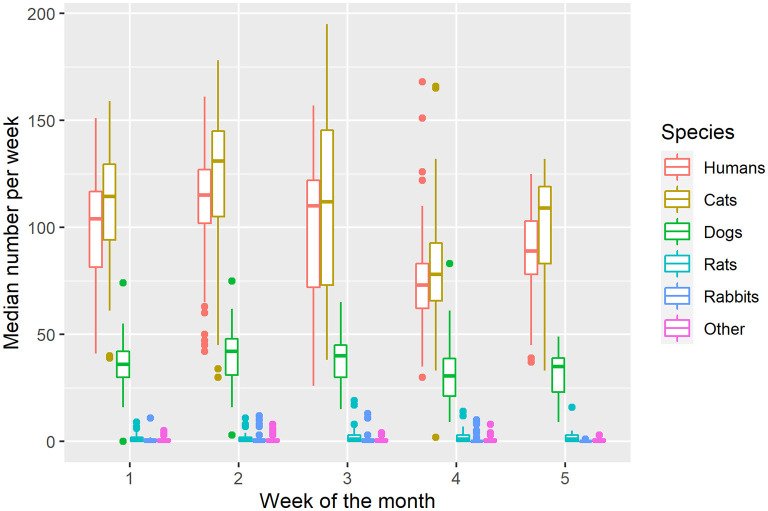
Median (IQR) number of clients per week by week of the month.

Human clients [H(4) = 55.9, *p* < 0.05], cats [H(4) = 51.7, *p* < 0.05], and dogs [H(4) = 26.8, *p* < 0.05] differed statistically significantly based on the week of the month. Rats [H(4) = 3.6, *p* = 0.47], rabbits [H(4) = 8.1, *p* = 0.09], and “other” animals [H(4) = 0.69, *p* = 0.95] did not differ based on the week (Figure 4).

The number of human clients serviced in Week 4 (median = 73, IQR = 20.75, *p* < 0.05) was lower than that of Weeks 1 (median = 104, IQR = 35.25, *p* < 0.05), 2 (median = 115, IQR = 25, *p* < 0.05), and 3 (median = 110, IQR = 50, *p* < 0.05), but equivalent to that of Week 5 (median = 89, IQR = 25, *p* < 0.05). The number of cats during Week 4 of each month (median = 78, IQR = 27, *p* < 0.05) was once again lower than that of Weeks 1 (median = 114.5, IQR = 35.25, *p* < 0.05), 2 (median = 131, IQR = 40, *p* < 0.05), and 3 (median = 112, IQR = 72.5, *p* < 0.05), but equivalent to that of Week 5 (median = 109, IQR = 36, *p* < 0.05; Figure 4).

Further, the same pattern was observed for dogs. The number of dogs during Week 4 of each month (median = 35, IQR = 16, *p* < 0.05) was lower than that of Weeks 1 (median = 36, IQR =12, *p* < 0.05), 2 (median = 42, IQR = 17, *p* < 0.05), and 3 (median = 40, IQR = 15, *p* < 0.05), but equivalent to that of Week 5 (median = 35, IQR = 16, *p* < 0.05; Figure 4).

The number of rats in Weeks 1 (median = 1, IQR = 2, *p* < 0.05), 2 (median = 1, IQR = 2, *p* < 0.05), 3 (median = 1, IQR = 3, *p* < 0.05), 4 (median = 1, IQR = 3, *p* < 0.05), and 5 (median = 1, IQR = 3, *p* < 0.05) were found to be equivalent. The same was also found for rabbits (medians = 0; IQR's = 1, 1, 1, 0, 0, for Weeks 1, 2, 3, 4, and 5 respectively, all *p* < 0.05), and “other” animals (medians = 0; IQR's = 1; *p* < 0.05 for all weeks; Figure 4).

## Discussion

### General Population

As a very rough estimate, the data showed that ~0.5% of the whole population of the DTES was serviced weekly through the pet food bank. As we suspected, the companion animal species served by the pet food bank in our target community, comprised of people who are experiencing hardship, but may be housed or partially housed, differed from the previous literature. We found that the companion animal composition in the DTES food bank service clientele was predominantly made up of cats. This is a surprising finding in this context, as previous research identified that the majority of companion animals owned by those experiencing homelessness tend to be dogs and not cats (13, 18, 35). The discrepancy may be explained by the ease of which dogs, as opposed to cats, can be maintained outdoors without a confined space (36, 37). The Charlie's food bank, however, not only services those who are experiencing homelessness, but also clients who live in low-income housing and have enclosed living quarters, which may explain this finding. The prevalence of small apartment units in the Downtown area (23) also helps explain the high proportion of cats owned, since it may be easier to keep cats in smaller spaces than dogs (36, 37). Our data highlight the need for researchers to evaluate programs serving a wider population to not make incorrect inferences about pet ownership.

Another result that warrants reflection is the size of the rat population represented, which is larger than that seen in the literature. The Animals Medicine Australia report (2019) (38) showed that 0.6% of companion animals owned were either mice or rats. In contrast, in our sample, we found that 1% of the companion animals were rats alone, and mice were a part of the “other” category. However, our data are consistent with that of the BC SPCA's shelter intake. A 5-year analysis of the BC SPCA animal population that services all of BC, revealed that rats make up a substantial proportion of admitted animals (39).

### COVID-19 Effects

The decrease in the number of clients serviced in 2020 was not unexpected and we interpret it as being due to the impacts of the COVID-19 pandemic. What is surprising, however, is the fact that the number of dogs, but not other pet species, present at the food bank each week was not statistically significantly different from that of the prior year. This could be explained in different ways. First, housing differences could account for the maintenance in dog numbers. As previously mentioned, owning dogs while living outdoors may generally be an easier experience than owning a cat outdoors (13, 18, 35–37). It could be the case that dog owners, who attend the food bank, are living outdoors more often than cat owners. This would mean that they would not have the option to stay at home despite the “stay at home orders,” and so would continue attending the food bank each week. Second, a possible explanation for this finding could be related to the price differences in owning a dog compared to a cat. Feeding prices for dogs generally tend to be higher than that for cats (40–42). This might then mean that dog owners are more dependent on the food bank and thus resistant to the public health recommendations to stay at home.

### Weekly Trends

We observed a decrease in the number of people as well as companion animals serviced at the food bank every fourth week of the month. That week (usually the last of the month) coincides with the time during which income assistance is distributed to many residents of the area (43, 44). This results in an increase in personal resource availability for clients, which in turn can decrease their need for assistance in pet food acquisition. This effect has been documented, for instance, in food purchasing practices, with people utilizing food stamps and increasing their caloric intake right after income supplementation checks are received (45–47).

The decrease in attendance at the pet food bank every fourth week of the month may also bring to light issues regarding access to care. It is possible that clients are prioritizing other needed purchases during that week and are unable to come for pet food assistance. Previous research identified that income assistance payments in the DTES population that struggles with drug addiction coincides with drug-related harms, albeit the phenomenon is highly complex and nuanced (26, 26, 48). With that in mind, one possible way to mobilize this data to action could be to consider increasing the services offered during the third week of each month, for instance by distributing extra food to clients, to overcome this potential barrier to care. This would ensure that the population is still supported throughout the month with no interruptions. The data also reveal that pet servicing is tightly related to community issues and cannot be adequately understood in isolation.

### Limitations

Our study focused on a limited population present in the Vancouver DTES area and uses data from a single pet food bank run by the BC SPCA. This decreases our ability to generalize our findings to other pet-owning populations experiencing financial strain in other areas of the world. However, unlike in previous studies, our data involves not only a population of people experiencing homelessness but also anyone who is experiencing financial hardship, providing a more comprehensive picture.

Further, we also missed 2 years of data (2015 and 2016), which could have provided us with a more complete understanding of the service patterns over time.

## Conclusion

We found that most companion animals serviced in the Downtown Eastside of Vancouver, B.C., Canada, each week were cats, followed by dogs. This surprising finding is likely due to the nature of our target population, which comprised of people experiencing homelessness as well as those that are housed or partially-housed. We also found that a large proportion of companion animals serviced were pet rats, which indicates a need for greater focus on supportive services for pet rats in pet food banks. Both of these findings highlight the need for researchers to increase their focus on more diverse populations when studying the human-animal bond and community services. Furthermore, we found that in 2020, the number of human clients, and cats, rats, rabbits, and “other” animals statistically significantly decreased from the previous year, likely due to COVID-19. However, the number of dogs serviced remained stable across time, suggesting that servicing needs may be different by pet species. Our data showed that attendance at the food bank was lower during the fourth week of each month, which coincided with income assistance schedules. This finding may bring to light access to care issues and highlight the need to consider pet food banks within the greater social services networks. Taking a “One Health” approach to servicing, that is, integrating provision of health and community support for humans and their pets (49), is likely to be a useful strategy. Future research is needed on efficacy and feasibility of merging human and companion animal servicing to the benefit of both.

## Data Availability Statement

The raw data supporting the conclusions of this article will be made available by the authors, without undue reservation.

## Ethics Statement

The studies involving human participants were reviewed and approved by The University of British Columbia Behavioral Research Ethics Board (H20-03807). Written informed consent for participation was not required for this study in accordance with the national legislation and the institutional requirements.

## Author Contributions

MS and AP contributed to the conception of the study, subsequent study design, and performed statistical analyses. MS acquired and organized the data and wrote the first draft of the manuscript. Both authors contributed to the manuscript, further revisions, and approved the final version of the manuscript for submission.

## Conflict of Interest

The authors declare that the research was conducted in the absence of any commercial or financial relationships that could be construed as a potential conflict of interest.

## Publisher's Note

All claims expressed in this article are solely those of the authors and do not necessarily represent those of their affiliated organizations, or those of the publisher, the editors and the reviewers. Any product that may be evaluated in this article, or claim that may be made by its manufacturer, is not guaranteed or endorsed by the publisher.
